# Case-control association between CCT-associated variants and keratoconus in a Saudi Arabian population

**DOI:** 10.1186/s12952-015-0029-5

**Published:** 2015-06-04

**Authors:** Khaled K. Abu-Amero, Inas Helwa, Abdulrahman Al-Muammar, Shelby Strickland, Michael A. Hauser, R. Rand Allingham, Yutao Liu

**Affiliations:** Department of Ophthalmology, College of Medicine, King Saud University, Riyadh, Saudi Arabia; Department of Cellular Biology and Anatomy, The Medical College of Georgia, Georgia Regents University, Augusta, GA USA; Departments of Medicine, Duke University Medical Center, Durham, NC USA; Department of Ophthalmology, Duke University Medical Center, Durham, NC USA

## Abstract

**Background:**

Keratoconus (KC) is the most common primary ectatic disease of the cornea and a major indication for corneal transplant. To date, limited KC-associated-risk loci have been identified. Association has recently been suggested between KC and 8 single nucleotide polymorphisms (SNPs) in the genomic regions of *FNDC3B*, *COL4A3*, *MPDZ-NF1B*, *RXRA-COL5A1*, *LCN12-PTGDS*, *FOXO1*, and *BANP-ZNF469*. These SNPs are associated with central corneal thickness (CCT), a known risk factor to KC. We are questioning whether these SNPs are significantly associated with KC in a Saudi Arabian population. The study included 108 unrelated KC cases and 300 controls. Patients were diagnosed with KC according to the Schimpff-flow based elevation map of the cornea. DNA genotyping was done using probe-based allelic discrimination TaqMan assays. Allele frequencies were compared between the cases and controls.

**Results:**

All SNPs were successfully genotyped with high efficiency (>95 %). The SNPs had no significant deviation in cases or controls from Hardy-Weinberg Equilibrium (HWE, p value > 0.05). None of the selected SNPs were significantly associated with KC in the Saudi Arabian population. However, we replicated the same trend of minor allele frequency (MAF) between cases and controls reported by a recent GWAS regarding the 5 SNPs rs4894535 (*FNDC3B*, chr3: 171995605), rs1536482 (*RXRA-COL5A1*, chr9: 137440528), rs7044529 (*COL5A1*, chr9: 137568051), rs11145951 (*LCN12-PTGDS*, chr9: 139860264), and rs2721051 (*FOXO1*, chr13: 41110884).

**Conclusions:**

This is the first study investigating the association of these SNPs with KC in a population from Saudi Arabia. We replicated the same trend of MAF alteration of the association between the SNPs rs4894535 (*FNDC3B*, chr3: 171995605), rs7044529 (*COL5A1*, chr9: 137568051), rs11145951 (*LCN12-PTGDS*, chr9: 139860264) and rs2721051 (*FOXO1*, chr13: 41110884) and KC-risk as reported by a recently published GWAS. Consistently replicated population-based studies are necessary to identify and/or confirm genetic susceptibility for certain diseases. We acknowledge that the lack of significance in our study is due to our small sample size and insufficient statistical power; however our data still add to the body of evidence of potential KC-candidate SNPs. This report aims at supporting the possible association between CCT-associated SNPs and KC susceptibility.

## Introduction

Keratoconus (KC) is the most common primary non-inflammatory ectatic disease of the cornea. It is one of the major indications for corneal transplant in the developed countries [1, 2]. KC usually develops during puberty or during the second decade of life. In most cases it progresses asymptomatically and both eyes are affected. The progression of the disease typically continues until the fourth decade of life before it eventually stabilizes [3]. The clinical signs of the keratoconus are highly variable depending on the stage of disease progression. Cases may present with any one or a combination of these different signs. Vogt’s striae can be observed by slit-lamp examination of the cornea. These striae appear as fine vertical lines in the deep stroma and Descemet’s membrane. They disappear transiently on gentle pressure. Munson’s sign is another useful feature for the diagnosis of keratoconus. This sign is observed as a v-shaped conformation of the lower lid when the eyelid is in a downgaze position. Munson’s sign develops as a result of corneal ectasia [2–4]. As the disease severely progresses, acute stromal edema occurs causing breaks in the Descemet’s membrane. These breaks in the membrane lead to aqueous leakage causing a phenomenon known as “hydrops” [5]. This stromal edema and breaks of the Descemet’s membrane further progress to corneal scarring. Despite the well-established clinical picture of keratoconus along with the extensive research on its etiology, the exact cause and pathogenesis remain largely unknown [2, 3, 6].

Increasing evidences suggest the involvement of a genetic component in disease susceptibility. This hypothesis is getting widely accepted especially with the predominance of KC among certain families, twins and particular ethnicities [7–9]. Accordingly, family history is an important risk factor and obtaining a thorough family medical history is crucial when considering a refractive surgery [8, 10]. Various candidate single nucleotide polymorphisms (SNPs) and copy number variations (CNV) have been associated with the susceptibility of KC [2, 6, 11–17]. In 2011, Rosenfeld et al. [18] reported copy number change (deletion) of 5q31 in KC patients. This group has reported the aforementioned CNV in a family with autosomal dominant KC as well as in patients with KC associated with other ocular and developmental abnormalities. The interest in CNV was not limited to the analysis of developmental pathway genes as in the case with deletions within 5q31. Recently, with the ongoing studies exploring the potential role of ROS in the pathogenesis of KC [19–21], a possible association between mtDNA (mitochondrial DNA) CNV and KC has also gained interest, yet still debatable. In 2015, Hao et al. [22] reported a significant decrease in mtDNA copy number in KC patients *versus* controls in Han Chinese population. This study further highlights the possible involvement of CNV in KC-susceptibility. Moreover, mtDNA may be an interesting candidate for further studies if we consider its direct contribution to mitochondrial functions and hence possible effects on the generation of reactive oxygen species (ROS). ROS is a known contributor to tissue damage and can be a significant player in the characteristic corneal thinning and ectasia that lead to the development and progression of KC [19].

Despite the available evidences and the extensive genetic research in KC over the past decades, the association of these loci with KC is still controversial and none of these genetic loci has been definitely confirmed as a KC-associated genetic factor [2, 11, 23–27]. Among the various reported KC-associated candidate genes are *VSX1* (Visual System Homeobox 1), *MIR184* (microRNA 184) and *DOCK9* (Dedicator of Cytokinesis 9), in addition to other candidate single nucleotide polymorphisms (SNPs) in various other genetic loci that need further investigations [13, 14, 16, 28]. On the contrary, many follow-up reports did not identify any mutations within these candidate genes in KC patients among the specific study populations [29, 30]. These contradictory data suggest the genetic heterogeneity of the disease. Accordingly, further extensive genetic analyses and wider genomic screening are necessary to identify the underlying genetic causation of the disease and to help understanding its uncertain pathogenesis.

Genome-wide association studies (GWAS) have become a powerful tool to investigate the genetic etiology of complex diseases [23]. Several GWAS have been performed to study KC in different populations. A study by Li and colleagues [24] detected no significant genome-wide associations in Caucasian KC patients. However, the most significant association was detected with variants near the *RAB3GAP1* gene whose mutation is also associated with other ocular abnormalities [31, 32]. Likewise, another report have suggested a possible association between KC and a variant related to *HGF* (hepatocyte growth factor) in an Australian population [33, 34]. Interestingly, the *HGF* gene has been previously reported in association with refractive error in Han Chinese and Caucasian populations [35, 36].

Corneal thinning is a known pathophysiological feature in KC and central corneal thinning has been previously reported as a major risk factor for KC [37, 38]. Recently, a meta-analysis including more than 20,000 European and Asian individuals investigated the association between central corneal thickness (CCT)-associated genetic variants and KC [15]. Of the 16 identified CCT-associated loci, only 2 loci conferred a significant association with KC. These loci were close to the *FOXO1* (forkhead box O1) and *FNDC3B* (fibronectin type III domain containing 3B) genes. Another study investigated the association between these 2 SNP variants and KC in an Australian population [39], but failed to replicate the reported association. However, they reported the same trend of minor allele frequency and odds ratio (OR) as the aforementioned report by Lu et al. [15]. Given the prerequisite of replicative studies to confirm the genetic associations of KC in different populations, we selected to investigate eight SNPs that have been previously reported in the GWAS by Lu et al. [15]. In this study, we attempt to investigate the association of these CCT-associated SNPs and KC in a population from Saudi Arabia.

## Results

Our study included 108 isolated KC cases and 300 controls matched for ethnicity. The average age for all KC subjects was 28.3 ± 6.9 years and 53.7 % of our cases were females. As shown in Table 1, the clinical examination revealed the presence of Munson’s sign in at least one eye of 43.3 % of the KC patients. Vogt’s striae was observed at least in one eye of 50.7 % of the KC patients. Corneal hydrops was noticed in at least one eye of 67.2 % of the KC patients while scaring was only detected in 29.9 % KC patients with at least one eye affected (Table 1). Average keratometry in KC patients was 51.1 ± 8.1D for the right eye and 53.2 ± 9.3D for the left eye. The average central corneal thickness in KC patients was 429.8 ± 82.3 μm for the right eye and 411.5 ± 86.8 μm for the left eye. Blood samples were collected from all subjects for DNA isolation and genotyping.

**Table 1:** Clinical phenotype of keratoconus patients

Mean age (years)		28.3 ± 6.9
% Female		53.7 %
% Munson Sign	OD	35.8 %
	OS	43.3 %
% Vogts Striae	OD	41.8 %
	OS	50.7 %
% Hydrops	OD	58.2 %
	OS	67.2 %
% Scarring	OD	20.9 %
	OS	29.9 %
Average Keratometry (In diopter)	OD	51.1 ± 8.1
	OS	53.2 ± 9.3
Central Cornea Thickness (μm)	OD	429.8 ± 82.3
	OS	411.5 ± 86.8

*OD* right eye; *OS* left eye

We selected the eight studied SNPs (Table 2) based on the study by Lu et al. [15]. These SNPs are located within or near the genomic regions of the following genes: *FNDC3B, COL4A3* (collagen type IV alpha 3), *MPDZ* (multiple PDZ domain protein)*-NF1B* (neurofibromin 1b), *RXRA* (retinoid X receptor alpha) *- COL5A1* (collagen type V alpha 1)*, LCN12* (lipocalin 12) *– PTGDS* (prostaglandin D2 synthase 21 kDa), *FOXO1* (Forkhead box protein O1), *and BANP* (BTG3 associated nuclear protein) *-ZNF469* (zinc finger protein 469). The selected SNPs are also associated with central corneal thickness (CCT), a known risk factor to KC. Genotyping call rate for rs9938149 (*BANP-ZNF49*, chr16:88331640), rs7606754 (*COL4A3*, chr2:228135180), and rs4894535 (*FNDC3B*, chr3:171995605) was 97.5 %, for rs7044529 (*COL5A1*, chr9:137568051) was 98.7, for rs2721051 (*FOXO1*, chr13: 41110884) was 99.1 %, for rs1536482 (*RXRA-COL5A1*, chr9:137440528) was 95.7, for rs1324183 (*MPDZ-NF1B*, chr9:13557491) was 97.3, and for rs11145951 (*LCN12-PTGDS*, chr9:139860264), was 99.5. The minor allele frequency (MAF) was calculated and compared in cases *versus* controls using 2x2 contingency tables. The significance level (p value) was calculated and odds ratio (OR) for each SNP was determined at a 95 % confidence interval (CI). The MAF, OR and p values for all the investigated SNPs in cases *versus* controls is summarized in Table 3.

**Table 2:** Summary of the screened SNPs, assays ID and probe sequences utilized in the study

dbSNP	Gene symbol	NCBI assembly location^a^	Assay ID	Context sequence [VIC/FAM]
rs7606754	*COL4A3*	chr2: 228135180	C__381076_10	AAATATACTTAGATCAAACTATTCA**[A/G]**AGAGGCTTGTTAAAATTTACCAATA
rs4894535	*FNDC3B*	chr3: 171995605	C__11808243_30	TTTTTTCCATTTTCCATAGTCCTTA**[C/T]**CCTTCCCTTTTGTTTTATATACCCA
rs1324183	*MPDZ-NF1B*	chr9: 13557491	C___7609107_10	GGTTTGTTTTTCACCCTATTCCTCC**[A/C]**ACTTCCTTCAAGGCCCCTTAGAACT
rs1536482	*RXRA-COL5A1*	chr9: 137440528	C___9201021_10	TAGGCTCCAACATGTAAATGCTGGG**[A/G]**GGACGCACACGTTCAAACCATAGCA
rs7044529	*COL5A1*	chr9: 137568051	C__29292365_20	TTTCCAGAAGTAACCCCTCAACTCT**[C/T]**TGATCGGTATTGTTCTGGACTTACT
rs11145951	*LCN12-PTGDS*	chr9: 139860264	C__31552589_20	AAGCTGCCAGTAGCTGCAGCTGGCC**[C/T]**GATGTGAGCCACAAAACCGGATGTG
rs2721051	*FOXO1*	chr13: 41110884	C__11302573_10	GAGCCAAATATCCTGCCAGCCAGCA**[C/T]**GGACCTGCTCCCAAGAATCATGTGT
rs9938149	*BANP-ZNF49*	chr16: 88331640	C__29917063_20	CTGCAATTGTCCCACAGGTCTTGTC**[A/C]**CCAGAAATCAAGTCCTTCTTCATCT

*SNP* single nucleotide polymorphism; *COL4A3* Collagen, type IV, alpha (Goodpasture antigen); *FNDC3B* fibronectin type III domain containing 3B; *MPDZ* multiple PDZ domain protein; *NF1B* neurofibromin 1b; *COL5A1* collagen, type V, alpha 1; *LCN12* lipocalin 12; *PTGDS* prostaglandin D2 synthase 21 kDa; *FOXO1* forkhead box O1; *BANP* BTG3 associated nuclear protein; *ZNF469* zinc finger protein 469

^a^The chromosomal location is based on UCSC GRCh37/hg19; chr, chromosome; Assays are supplied by Life technologies (Carlsbad, CA, USA)

**Table 3:** Association between CCT-associated SNPs and KC risk in the Saudi Arabian study population

Locus	Lead SNP	A1/A2	MA	MAF Case	MAF Control	*p*-value	OR (95 % CI)
*COL4A3*	rs7606754	A/G	A	0.35	0.37	0.6	0.9 (0.7-1.3)
*FNDC3B*	rs4894535	C/T	T	0.16	0.12	0.1	1.4 (0.9-2,2)
*MPDZ-NF1B*	rs1324183	A/C	A	0.25	0.28	0.4	0.8 (0.8-1.2)
*RXRA-COL5A1*	rs1536482	A/G	A	0.45	0.41	0.4	1.1 (0.8-1.6)
*COL5A1*	rs7044529	C/T	T	0.14	0.13	0.8	1.1 (0.7-1.7)
*LCN12-PTGDS*	rs11145951	C/T	T	0.44	0.49	0.2	0.8 (0.6-1.1)
*FOXO1*	rs2721051	C/T	T	0.14	0.11	0.2	1.4 (0.9-2.2)
*BANP-ZNF49*	rs9938149	A/C	C	0.29	0.32	0.4	0.9 (0.6-1.2)

*CCT* Central cornea thickness; *SNP* single nucleotide polymorphism; *KC* keratoconus; *A1/A2* Allele 1/Allele 2; *MA* minor allele; *MAF* minor allele frequency; *p*-value, derived from two-tailed fisher exact test; *OR* odds ratio; *CI* confidence interval. *COL4A3* Collagen, type IV, alpha (Goodpasture antigen); *FNDC3B* fibronectin type III domain containing 3B; *MPDZ* multiple PDZ domain protein; *NF1B* neurofibromin 1b; *COL5A1* collagen, type V, alpha 1; *LCN12* lipocalin 12; *PTGDS* prostaglandin D2 synthase 21 kDa; *FOXO1* forkhead box O1; *BANP* BTG3 associated nuclear protein; *ZNF469* zinc finger protein 469

Our data did not show any statistically significant association of the eight previously reported SNPs with KC in the Saudi Arabian population. However, in comparison to the GWAS by Lu et al*.* [15], we did notice a similar trend/magnitude of allele frequency alterations between cases and controls for 5 of the 8 investigated SNPs (Table 4), including rs4894535 (*FNDC3B*, chr3:171995605), rs1324183 (*MPDZ-NF1B*, chr9:13557491), rs1536482 (*RXRA-COL5A1*, chr9:137440528), rs2721051 (*FOXO1*, chr13: 41110884) and rs9938149 (*BANP-ZNF49*, chr16:88331640). Based on the Quanto calculation, our dataset had a relatively low statistical power to detect significant associations, ranging from 20–64 %. We noticed that SNPs rs4894535 (*FNDC3B*, chr3:171995605) and rs2721051 (*FOXO1*, chr13:41110884) yielded the highest statistical power of 50 % and 64 % respectively for a positive association.

**Table 4:** Comparison between MAF in our data set and the data set from Lu et al. 2013 [15] Nat Genetics

SNP and MA			Data from the this study			Data from Lu et al. 2013 [15]		
			(108 cases/300 controls)			(652 cases/2,761 controls)		
Lead SNP	Allele	MAF (cases)	MAF (controls)	OR	MAF (cases)	MAF (controls)	OR	*p*-value
rs7606754	A	0.34	0.37	0.9	0.37	0.33	1.2	6.0E-03
rs4894535^a^	T	0.16	0.12	1.4	0.21	0.16	1.5	1.3E-06
rs1324183	A	0.25	0.28	0.9	0.24	0.2	1.3	8.8E-05
rs1536482	A	0.45	0.41	1.1	0.4	0.34	1.3	1.2E-03
rs7044529^a^	T	0.14	0.13	1.1	0.18	0.14	1.3	3.0E-04
rs11145951^a^	T	0.44	0.49	0.82	0.45	0.49	0.89	0.03
rs2721051^a^	T	0.14	0.11	1.4	0.14	0.1	1.5	3.2E-06
rs9938149	C	0.29	0.32	0.9	0.32	0.36	1.2	7.1E-03

*SNP* single nucleotide polymorphism; *MA* minor allele; *MAF* minor allele frequency; *OR* odds ratio

^a^ These SNPs had similar trends of allele frequency changes between cases and controls as those reported by Lu et al. [15]

## Discussion

This is the first report suggesting an association between CCT-associated SNPs and KC in a population from Saudi Arabia. Population-based replicative studies are necessary to identify and/or confirm genetic susceptibility for certain diseases. We present a cohort of homogenous ethnic background and well-defined clinical phenotype in an attempt to confirm the genetic association reported by a recent CCT GWAS by Lu et al. [15]. Their study was conducted among individuals with European and Asian ancestries and identified significant associations between KC and CCT in a meta-analysis of more than 20,000 individuals including KC-affected and non-affected individuals. They further examined the association between these CCT loci and KC risk in two independent studies that include samples from 2 separate populations (Australian and European American). Among CCT-associated SNPs, this group reported an association between KC and five CCT-associated SNPs. In the aforementioned report only two of the five reported SNPs; rs2721051 (*FOXO1*, chr13:41110884) and rs4894535 (*FNDC3B*, chr3:171995605), actually achieved statistical significance in association with KC with p-values of 3.2E-06 and 1.3E-06 respectively and with an OR of 1.5 for both SNPs. Although, our study was unable to detect a statistically significant association between the reported SNPs and KC, our data were still consistent in the effect direction as compared to the study by Lu et al. [15].

Furthermore, similar to the aforementioned GWAS, we consistently replicated the same minor alleles in cases and controls for all SNPs and the same trend of OR and MAF alterations in five of the eight suggested KC-associated alleles. Moreover, comparing our results to those reported by Lu et al. [15], both studies reported almost the same minor allele frequency for 4 of the reported eight alleles namely, rs4894535 (*FNDC3B*, chr3:171995605), rs1324183 (*MPDZ-NF1B*, chr9:13557491), rs2721051 (*FOXO1*, chr13:41110884) and rs9938149 (*BANP-ZNF49*, chr16:88331640) whereas the remaining four SNPs share the same trend of MAF although the frequency does not exactly match (Table 4). As for the OR, our data show that SNP rs4894535 (*FNDC3B*, chr3:171995605) and rs2721051 (*FOXO1*, chr13:41110884), has an OR of 1.4, which is close to the OR of these SNPs from Lu et al. [15] (OR, 1.5). These SNPs are also interestingly the ones that achieved significant association with KC risk in the GWAS by the same group [15].

Accordingly, our study has achieved the same trend of KC-association of the SNPs reported by Lu et al.; however, our small sample size (108 cases and 300 controls) is probably the main reason behind the lack of significance and the limited statistical power (20 %–64 %) of our dataset. Despite these facts, there is still a clear overlap between our data set and that of Lu et al. especially with the SNPs achieving most significance in their study which are rs4894535 (*FNDC3B*, chr3:171995605) and rs2721051 (*FOXO1*, chr13:41110884).

In the same context, Sahebjada and colleagues [39] have also attempted to study the association between KC and five of these CCT-associated SNPs which include rs1324183 (*MPDZ-NF1B*, chr9:13557491), rs1536482 (RXRA-COL5A1, chr9:137440528), rs2721051 (*FOXO1*, chr13:41110884), rs7044529 (*COL5A1*, chr9:137568051) and rs9938149 (*BANP-ZNF49*, chr16:88331640). Similarly, with a sample size of 157 KC cases and 673 controls, their dataset was not able to achieve statistical significance in association with KC at any of these SNPs. Similar to ours, their data failed to detect an association between SNPs rs1536483 and rs7044529 and KC risk with OR = 1.1. Our study was unable to replicate the suggested association by Sahebjada et al. [39] between SNPs rs1324183 (*MPDZ-NF1B*, chr9:13557491) (OR = 1.68) and rs9938149 (*BANP-ZNF49*, chr16:88331640) (OR = 1.47) with KC. Despite the non-significant results in both studies, our data and the study by Sahebjada et al. [39] indicated the consistent trend of MAF changes that Lu and colleagues [15] have reported.

Interestingly, a recent publication by Hao et al. [40] also aimed at replicating the association between KC and six of the SNPs we assessed. Similar to ours, their report studied SNPs rs4894535 (*FNDC3B*), rs1324183 (*MPDZ-NF1B*), rs1536482 (RXRA-COL5A1), rs7044529 (*COL5A1),* rs2721051 (*FOXO1)* and rs9938149 (*BANP-ZNF49).* Hao and coworkers [40] successfully reported a significant association with rs1324183 (*MPDZ-NF1B*) in a cohort of 210 sporadic KC patients from a Han Chinese population. The MAF and OR reported by their group was 0.288 and 3.1 respectively. Compared to the previous reports for the SNP rs1324183 (*MPDZ-NF1B*), Lu et al. reported a MAF and OR of 0.24 and 1.33 respectively, and Sahebjada et al. reported a MAF and OR of 0.29 and 1.68 respectively. Consistently, our study reported a MAF of 0.25, however the OR for this SNP in our cohort was in the range of 0.8-1.2 probably due to our sample size which is small compared to all the above-mentioned studies. Furthermore, the inconsistency regarding the OR and significance level across the four studies is mainly due to the wide variation in the sample size: the study by Lu et al. [15] was conducted with 652 cases and 2,761 controls, that of Sahebjada et al. [39] with 157 cases and 673 controls, that of Hao et al. [40] with 210 cases and 191 controls, and our study with 108 cases and 300 controls. However, the similar MAF and OR trend confers that the SNP rs1324183 (*MPDZ-NF1B*) may be associated with KC in a small percentage of KC cases and its contribution may vary among populations. Taken together, our data as well as others’ suggest following up on these CCT-associated SNPs and KC by additional replicative large scale studies within different populations.

In contrast, the other SNP that was reported by Hao et al. [40] with a borderline significance in the Han Chinese population (rs295654; *LOX*) was not examined by any of the other three reports. Nevertheless, *LOX* gene has been previously reported as a candidate gene for KC [41], thus although we are not able to contrast their results to the findings by other groups, we believe that further population-based follow-up studies regarding this SNP may reveal a relevant genetic association.

## Conclusions

In summary, we replicated the same trend of MAF alteration and the suggested association between the SNPs rs4894535 (*FNDC3B*, chr3: 171995605), rs7044529 (*COL5A1*, chr9: 137568051), rs11145951 (*LCN12-PTGDS*, chr9: 139860264) and rs2721051 (*FOXO1*, chr13: 41110884) and KC-risk as reported in the GWAS by Lu et al., the Australian study cohort by Sahebjada et al. [39] as well as the most recent Han Chinese study by Hao et al. [40]. We understand that the small sample size of our data set is a major weakness in this study, however we sought to report the consistency of our results in supporting previously published population-based studies. Our future plan is to recruit more KC cases from the Saudi Arabian population in an attempt to increase the statistical power of our study and further confirm the existing results. We believe that our replicative genetic study still supplements the body of evidence available through population-based GWAS.

## Methods

### Study population

This case–control study adheres to the tenets of the Declaration of Helsinki and was approved by the College of Medicine ethical committee at King Saud University (Riyadh, Saudi Arabia). All participants signed an informed consent. Study subjects were self-identified as Saudi Arabians furthermore; their ethnicity was confirmed through database of Arab families of Saudi Arabian origin. Male and female patients (n = 108) were recruited from the anterior segment clinic at King Abdulaziz University after examination. Cases and controls were selected according to the inclusion and exclusion criteria described previously [11, 42, 43]. In brief, KC cases are diagnosed with the Schimpff-flow based examination and are included in the study if the Schimpff-flow–based elevation map showed posterior corneal elevation within the central 5 mm ≥ +20 μm, inferior-superior dioptric asymmetry (I-S value) > 1.2 diopters (D), and the steepest keratometry > 47D. Cases were thoroughly examined for the presence of keratoconus common clinical features including Munson’s sign, Vogt’s striae, hydrops and scaring. Keratometry was also measured for both eyes and summary of the clinical phenotype for all cases is included in Table 1. Thorough family history is obtained for each patient and only one member from each family is included in the study. On the other hand, sporadic cases were identified after examining their immediate family members and accordingly were confirmed as isolated cases of KC. The control subjects (n = 300) were recruited from the general ophthalmology clinic. Controls were selected if slit-lamp exam showed clear cornea and Schimpff-flow–based elevation map within the normal limit. Exclusion criteria for KC cases included refusal to participate, post-LASIK ectasia as well as secondary KC cases including those secondary to trauma, surgery, Ehlers-Danlos syndrome, osteogenesis imperfecta, and pellucid marginal degeneration, while exclusion criteria for control subjects included detection of any ocular disease(s) or previous ophthalmic surgeries.

### DNA genotyping

DNA was isolated from peripheral blood samples as described previously [42–44]. Briefly, DNA was extracted from the buffy layer using the Illustra blood genomicPrep Spin kit (GE Healthcare Life Sciences, Buckinghamshire, UK). We selected to screen eight CCT-associated SNPs based on a study by Lu et al. [15]. These SNPs included rs7606754 (*COL4A3*, chr2:228135180), rs7044529 (*COL5A1*, chr9:137568051), rs4894535 (*FNDC3B*, chr3:171995605), rs2721051 (*FOXO1*, *chr13: 41110884*), rs1536482 (*RXRA-COL5A1*, chr9:137440528), rs1324183 (*MPDZ-NF1B*, chr9:13557491), rs11145951 (*LCN12-PTGDS*, chr9:139860264), and rs9938149 (*BANP-ZNF49*, chr16:88331640). The selected SNPs are related to different genetic loci as shown in Table 2. TaqMan assays from Life Technologies (Carlsbad, CA, USA) were used and their sequences are shown in Table 2. TaqMan allelic discrimination assays were employed for genotyping these 8 SNPs by the use of Assays-On-Demand products with the ViiA7 Realtime PCR system with 384-well block according to the standard protocols from the manufacturer (Life Technologies Carlsbad, CA, USA). All 8 SNPs were genotyped in 408 Saudi Arabian DNA samples. For quality control (QC) purposes, two CEPH (the Centre d'Etude du Polymorphisme Humain, Foundation Jean Dausset, Paris, France) standards were included in each 96-well plate, and samples from two individuals were duplicated across all plates, with the laboratory technicians masked to their identities. Analysis of genotypes required matching QC genotypes within and across plates and achieving a genotyping efficiency of at least 95 %.

### Statistical analysis

Hardy-Weinberg equilibrium (HWE) test of all SNPs was performed using Michael H. Court’s (2005–2008) online calculator (https://www.google.com/webhp?sourceid=chrome-instant&ion=1&espv=2&ie=UTF-8#q=Hardy-Weinberg+equilibrium+Tufts+online+calculator). All statistical analyses were performed using JavaStat (http://statpages.org/ctab2x2.html) with 2-by-2 contingency tables [45]. *P*-values were calculated using two-tailed Fisher exact test and odds ratio (OR) was derived with 95 % confidence interval (CI) using JavaStat [45]. Power calculations were performed using Quanto software version 1.2.4 (University of Southern California, Los Angeles, CA) using previously described methods, assuming a population prevalence of 0.05 % and a log-additive risk model [46, 47]. The power for each SNP was calculated using the previously reported OR in Lu et al. [15] and the allele frequency in our Saudi Arabia controls.

## Abbreviations

SNP: Single nucleotide polymorphism
KC: Keratoconus
CCT: Central corneal thickness
HWE: Hardy-Weinberg Equilibrium
GWAS: Genome-wide association study
CNV: Copy number variation
ROS: Reactive oxygen species
*FNDC3B*: Fibronectin type III domain containing 3B
*COL4A3*: Collagen type IV alpha 3
*MPDZ*: *M*ultiple PDZ domain protein
*NF1B*: Neurofibromin 1b
*RXRA*: Retinoid X receptor alpha
*COL5A1*: *C*ollagen type V alpha 1
*LCN12*: Lipocalin 12
*PTGDS*: Prostaglandin D2 synthase 21 kDa
*FOXO1*: Forkhead box protein O1
*BANP*: BTG3 associated nuclear protein
*ZNF469*: Zinc finger protein 469
*VSX1*: Visual System Homeobox 1
*MIR184*: MicroRNA 184
mtDNA: Mitochondrial DNA
*DOCK9*: Dedicator of Cytokinesis 9
*HGF*: Hepatocyte growth factor
LOX: Lysyl oxidase
MAF: Minor allele frequency
OR: Odds ratio
CI: Confidence interval
D: Diopters
